# The *Neurospora crassa* Pangenome: A Robust Framework for Population-Scale Analysis and Structural Variant Discovery

**DOI:** 10.3390/jof12070507

**Published:** 2026-07-09

**Authors:** Huawei Tan, Sihai Yang, Xiaohui Zhang

**Affiliations:** School of Life Sciences, Nanjing University, Nanjing 210023, China

**Keywords:** comparative genomics, phylogenetic analysis, pan gene family, stress-related genes, transposons

## Abstract

*Neurospora crassa* is a widely distributed ascomycete with high genetic diversity, yet reliance on limited reference genomes has hindered a comprehensive understanding of its genetic landscape. To address this limitation, we integrated the functional annotation of the FGSC2225 genome with a comprehensive comparative genomic analysis of *N. crassa* strains. FGSC2225 gene and transposable element (TE) proportions mirrored those of FGSC2489, though TE levels were significantly higher than those in sister species *Sordaria macrospora*. Phylogenetic analysis resolved the *N. crassa* population into two primary lineages: Clade A (including FGSC2489 and FGSC2225) and Clade B (including FGSC4830), with the former exhibiting larger genome sizes. Leveraging de novo assemblies of 72 high-quality draft genomes, we constructed a comprehensive pangenome to investigate the molecular evolution of various gene families. For example, systematic phylogenetic analysis of the HET-domain-containing gene family and three stress-related families—heat shock transcription factor, basic leucine zipper, and Cytochrome P450—demonstrated varying degrees of conservation and presence/absence variation across the lineages. Addressing the limitations of current genomic resources, this work provides a pangenomic framework to detect rapid adaptive evolution in filamentous fungi. This methodology serves as a robust template for identifying transcription factors, effectors, and structural variations critical to stress response and virulence in diverse fungi.

## 1. Introduction

*Neurospora crassa* is a filamentous fungus belonging to the phylum Ascomycota and is widely distributed in nature. As a classic model organism in genetics and molecular biology, *N. crassa* has an extensive research history. Its significant advantages include a haploid life cycle, a relatively small genome of approximately 41 Mb, rapid growth and reproduction, and an efficient genetic manipulation system. These characteristics established it as an ideal material for biochemical and genetic research as early as the beginning of the 20th century. Most notably, Beadle and Tatum proposed the famous one gene-one enzyme hypothesis through their work on *N. crassa*. This research laid the foundation for molecular genetics and resulted in the 1958 Nobel Prize in Physiology or Medicine [1,2]. In recent years, although the rise in medical and agricultural applications has shifted research focus toward human and plant pathogenic fungi [3], *N. crassa* remains a fundamental model for studying genomic evolution and adaptive conflict. As one of the filamentous fungi with the highest levels of genetic variation in natural populations, its unique Repeat-Induced Point mutation (RIP) mechanism provides a distinct lens through which to examine these processes [4].

Evolutionary studies of *N. crassa* have historically relied almost exclusively on the FGSC2489 reference genome. However, substantial intra-specific genetic divergence remains under-explored. A primary example is the FGSC2225 (Mauriceville-1cA) strain, a natural strain collected from Mauriceville, Texas, USA [5]. This strain is genetically distinct from the reference strain FGSC2489, with its genome exhibiting ~1.82% sequence divergence. Given the paucity of fully sequenced genomes for this species, current data fail to capture the full spectrum of its global genetic diversity. To address this gap, recent work in our laboratory produced a telomere-to-telomere (T2T) level genome assembly for the FGSC2225 strain. In the present study, we perform a comparative analysis of the genome structure, functional gene repertoire, and transposable element landscape between FGSC2225 and other representative strains.

Recent genomic studies of multiple individuals from the same species have uncovered large differences in the gene content between individuals. It has become increasingly common to refer to species with multiple genomes sequenced in terms of their ‘pan-genome’ [6]. The pan-genome is the union of ‘core’ conserved genes and all ‘accessory’ non-conserved genes across all strains of a species. Species pan-genomes have been analyzed in many prokaryotic, plants, and fungi. In fungi, a number of studies of the *Saccharomyces cerevisiae* pan-genome, including a recent large-scale analysis of genome evolution across 1011 strains, have shown evidence for an accessory genome of varying size, as well as large variation in subterminal regions across multiple *S. cerevisiae* strains [7]. Here, we have investigated the pan-genomes of model fungal species *N. crassa* by integrating 76 draft genomes of *N. crassa* strains. Our efforts aimed to explore the divergent lineages of *N. crassa* more extensively, providing valuable resources for future molecular research.

## 2. Materials and Methods

### 2.1. Genome Annotation

The *N. crassa* strain FGSC2225 (mat-A) was kindly provided by Shaojie Li, State Key Laboratory of Mycology, Institute of Microbiology, Chinese Academy of Sciences, China. A draft genome for *N. crassa* FGSC2225 was built using PacBio RSII subreads, and then assembled by Canu software (Details described in another in-review paper, https://zenodo.org/records/20659372, accessed on 19 January 2026). We performed whole-genome alignment between *N. crassa* FGSC2225 and other strains using MUMmer (v4.0) [8] with the parameters “nucmer --maxmatch -c 100 -b 500 -l 50”. Structural variations and polymorphism were subsequently identified using Syri (v1.7) [9].

Genome-wide identification of TEs in the FGSC2225 genome was performed using a combination of de novo and homology-based approaches [10,11,12]. The gene was predicted by the BRAKER3 [13] and the EVM program [14] with RNA evidence. We performed tRNA prediction using tRNAscan-SE (v2.0.7) [15] with default parameters. The identification of ribosomal RNAs (rRNAs) was conducted using barrnap (v0.9) with the following specific parameters: --kingdom euk –lencutoff 0.8 –evalue 1e-6. For other non-coding RNAs (ncRNAs), sequences were searched and classified against the Rfam database [16]. The OrthoFinder software (v2.5.5) was employed for the identification of orthologs [17]. The Ka/Ks in orthologous gene pairs was calculated with ParaAT (v2.0) and KaKs_Calculator (v2.0) programs [18,19].

### 2.2. Phylogenetic and Population Genetics Analysis

The reference genomes and re-sequencing data were downloaded following previous reports [20,21,22], Mycocosm Database (https://mycocosm.jgi.doe.gov/fungi, accessed on 19 January 2026) and NCBI SRA database. During the variant calling, the *N. crassa* strains’ re-sequencing reads were aligned to FGSC2225 using the BWA-MEM aligner (0.7.10-r789) [23]. Variant calling was performed using GATK (v3.7) with both the HaplotypeCaller and UnifiedGenotyper algorithms [24], and then were integrated into a consolidated VCF file. The potential variants were filtered with GATK: VariantFiltration for SNPs (QD < 2, QUAL < 30, SOR > 3, FS > 60, MQ < 40, MQRankSum < −12.5, ReadPosRankSum < −8) and InDels (QD < 2, QUAL < 30, FS > 200, ReadPosRankSum < −20).

Prior to population genetic analyses, variants were filtered using PLINK (v1.9) [25]. As each branch contained at least 3 *N. crassa* strains, the variations dataset was pruned with a minor allele frequency (MAF) threshold of ≥3% (≥3 samples). A total of 100,000 loci were randomly subsampled to resolve the phylogenetic relationships of 100 *N. crassa* strains, and then the phylogenetic tree was constructed by using IQ-TREE software (v2.1) [26] with the parameters “-model TEST -b 1000”. Subsequently, *N. crassa* population structure was assessed via Principal Component Analysis (PCA) using PLINK (v1.9) [25], with a decomposition dimension of 100. Loss-of-function (LoF) variants were identified, including stop-gain mutations, nonsynonymous SNVs, and frameshift indels [27].

### 2.3. De Novo Assembly of Genomes by Next-Generation Data

The genomes of 96 strains were de novo assembled using the MaSuRCA (v4.1.0) [28] based on high-depth resequencing data. To filter sequences from non-target species, Kraken2 [29] was employed with the pluspfp database (v20251015) to identify and remove reads classified as bacterial or human contaminants. Subsequent coding gene prediction was conducted using BRAKER3 [13], following previously described parameters to ensure accurate functional annotation.

### 2.4. Construction of Pangenome

For the graph-based pangenome, FGSC2489 served as the reference backbone. SNPs and small indels were identified using the show-snps command (-ClrT) following nucmer alignment (--maxmatch -c 90 -l 40) and then subsequent filtering with delta-filter (-1 -i 90 -l 100) [8]. To capture larger genomic changes, non-redundant structural variations (SVs) from the FGSC2225, FGSC73, and FGSC4830 genomes were integrated into a consolidated VCF file. In addition to the SNP and indel pipeline, we utilized SyRI (v1.7) [9] to automate the discovery of larger insertions and deletions (INS/DEL) as SVs. The final variation graph was then constructed using the vg toolkit (v1.70) [30].

To further characterize the *N. crassa* pangenome, except for the abovementioned 4 chromosome-level genomes, we utilized the four chromosome-level genomes and 72 high-quality assemblies (N50 > 100 kb) for comprehensive variant detection and gene content analysis. Orthologous gene groups were identified across all strains using OrthoFinder (v2.5.5) [17]. Based on gene family occupancy, we categorized the pangenome into four distinct sets: core gene families (present in all strains), softcore families (missing in only one strain), dispensable families (present in two or more strains but missing in more than one), and private families (unique to a single strain).

### 2.5. Identification of Gene Families

The Pfam protein family database (release 38.2) was downloaded from the EBI repository (https://ftp.ebi.ac.uk/pub/databases/Pfam/releases/Pfam38.2/Pfam-A.hmm.gz, accessed on 21 April 2026) [31]. To identify gene families across the pangenome, we searched all predicted proteomes against the Pfam Hidden Markov Models (HMMs). Candidates were filtered using an E-value threshold of <1e-4 and a minimum domain alignment coverage of 50%. Presence–absence variations (PAVs) for these gene families were assessed across the 76 *N. crassa* strains. Based on the PAV data, we characterized the expansion and contraction dynamics of each gene family by calculating the mean member number, the coefficient of variation (CV), and the Fano factor (F). These metrics were defined as follows: (1) Mean member number: The average number of gene copies per family across all 72 strains; (2) Coefficient of Variation (CV): Used to represent the relative dispersion of gene family size, calculated as the ratio of the standard deviation to the mean (CV = standard deviation/mean); Fano factor (F): Used to measure the clumping or overdispersion of gene family distributions, calculated as the ratio of the variance to the mean (F = variance/mean).

Specifically, we extracted and analyzed the Heterokaryon incompatibility proteins (HET; PF06985.18 and PF07217.17), heat shock transcription factors (HSFs; PF00447), basic leucine zippers (bZIPs; PF00170), and Cytochrome P450s (CYP450s; PF00067). Multiple sequence alignments of the domains in each gene family were performed using MUSCLE v3.8 [32]. Neighbor-joining phylogenetic trees were subsequently constructed in MEGA v11 using the p-distance model, pairwise deletion of gaps, and 1000 bootstrap replicates.

Furthermore, we calculated the ratio of non-synonymous to synonymous substitution rates, Ka/Ks, as previously described. Genes exhibiting Ka/Ks > 1 were identified, and the proportion of such genes was calculated for each strain. Finally, a heatmap was generated in R to visualize the clustering of both strains and genes based on their Ka/Ks > 1 profile.

## 3. Results

### 3.1. FGSC2225 Genomic Resources

The T2T-level assembly of the *N. crassa* FGSC2225 genome spans approximately 41 Mb and comprises seven gapless nuclear chromosomes alongside a complete mitochondrial genome (In another paper, in review). Protein-coding genes for the FGSC2225 genome were predicted using a combination of multiple ab initio gene prediction software. A total of 9726 genes were predicted, of which 9699 (99.72%) FGSC2225 proteins showed homology with proteins within the *Neurospora* genus. The total length of coding genes was ~16.79 Mb in FGSC2225, representing 40.7% of the total genome sequence. Gene lengths range from 75 bp to 33,595 bp, with an average length of 1726 bp, which includes an average CDS length of 1474 bp and an average intron length of 252 bp. The genes are arranged relatively compactly in the nuclear genome, with most located in non-repetitive regions and a median intergenic distance of 1216 bp (Table S1).

A total of 7012 coding genes in the *N. crassa* FGSC2225 genome were annotated using the Swiss-Prot, UniProt, InterProScan, eggNOG-mapper, and NR databases (Figure S1). This effort resulted in the identification of approximately 3000 more protein-coding genes than the FGSC2489 NC12 version [20]. Furthermore, we also predicted and annotated 602 ncRNAs in *N. crassa* FGSC2225, including 118 rRNAs, 446 tRNAs, 10 snRNAs, 17 snoRNAs, and 11 other non-coding RNAs (Table S2).

### 3.2. Genomic Comparison Between Strains FGSC2225, FGSC2489, and FGSC4830

**Figure 1:**
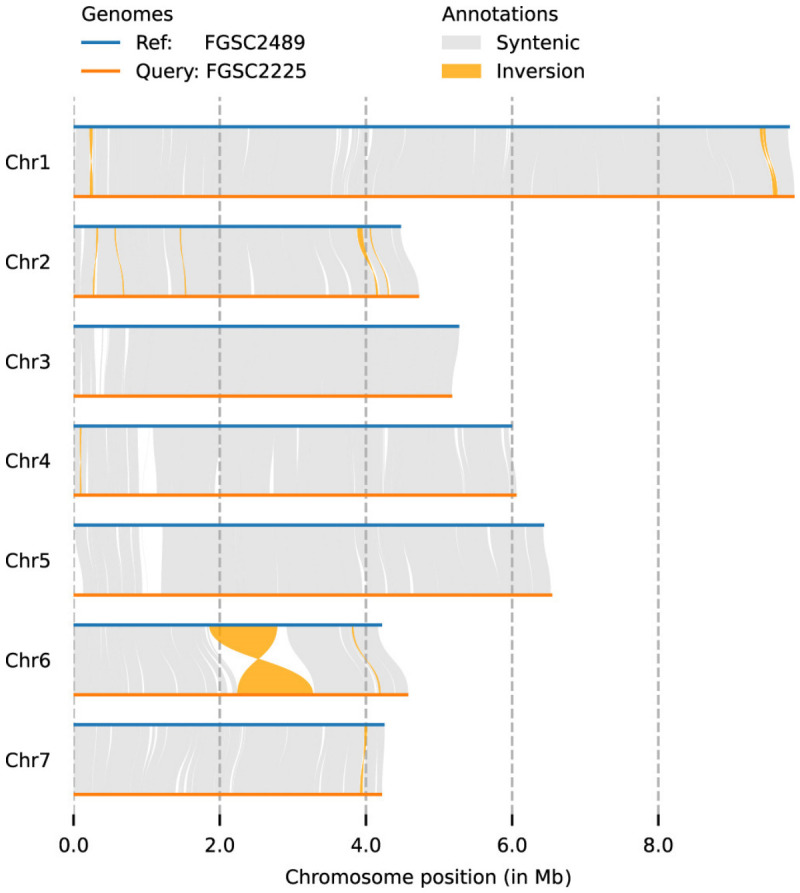
Whole-genome collinearity analysis of *Neurospora crassa* FGSC2489 (Ref) and FGSC2225 (Query). Genomic alignment was performed using Mummer4, and structural variants were identified and classified using SyRI v1.7. The visualization was generated using plotsr.

To investigate whether FGSC2225 has unique genetic characteristics, we performed comparative genomic analysis with the most well-studied strain, FGSC2489. First, we identified 8352 gene clusters, comprising 9006 individual genes, that were present exclusively in the FGSC2225 genome and absent in FGSC2489. While a significant portion of these unique genes represent proteins of unknown function, others include those involved in cytochrome oxidase activity, RNA-binding, DNA recombination and repair (Table S3). Second, compared with strain FGSC2489 (180 bp), strain FGSC2225 has a larger size of introns (252 bp). Third, a total of 18 gene families, containing 53 genes, were found to be more abundant in FGSC2225 than in FGSC2489. In particular, the number of genes related to alcohol dehydrogenase in strain FGSC2225, with four copies (NCG01754, NCG02476, NCG20009, and NCG20543), was two more than the count identified in strain FGSC2489 (Table S4). Fourth, both strains shared 325 conserved syntenic blocks, which accounted for 92.8% (37.58 Mb) and 91.9% (36.84 Mb) of the genome size in FGSC2225 and FGSC2489, respectively. We identified 20 inversions, including a prominent large-scale inversion situated in Chr6 (Figure 1, Table S5). At the sequence level, the strains displayed 499,213 SNPs alongside substantial small-scale variation, including 52,663 insertions (1.73 Mb) and 48,283 deletions (1.44 Mb). Fifth, out of 8969 collinear genes, 258 orthologous genes (2.8%) exhibit a Ka/Ks ratio greater than 1 when comparing FGSC2225 to FGSC2489.

In comparison to the FGSC2489 reference genome, we identified 30 strain-specific genes unique to FGSC2225. Additionally, 83 genes were found to be completely absent in FGSC2225, while another ~500 genes exhibited partial deletions or substantial sequence variation. In addition to PAV, frequent instances of intron gain, loss, and sequence polymorphism were observed among *N. crassa* strains. Although fundamental genomic attributes, such as total gene length and CDS length, remain largely consistent between the two strains, distinctive disparities emerged in their intron distribution patterns. Specifically, the proportion of intron-containing genes in FGSC2225 is significantly higher than in FGSC2489, whereas the prevalence of single-exon genes is correspondingly low (Figure S2a). Further investigation identified a substantial number of intronic insertion/deletion variants within orthologous gene pairs. Out of 8868 orthologous gene pairs between FGSC2489 and FGSC2225, 1173 (13.2%) coding genes in FGSC2225 show more introns than in FGSC2489; 440 (5.0%) genes contain fewer introns, and 7255 (81.8%) genes keep the same intron number (Figure S2b). This dynamic structural variation results in marked differences in intron counts for orthologs across different strains, underscoring the considerable evolutionary plasticity of intronic architecture within the *Neurospora* genome.

Comparative genomic analysis revealed more pronounced divergence between strains FGSC2225 and FGSC4830. Notably, the genome of FGSC2225 (41.10 Mb) is significantly larger than that of FGSC4830 (37.06 Mb). Analysis of gene content identified 8205 gene clusters (comprising 8738 individual genes) that lack orthologs in FGSC4830; this represents a higher degree of variation than the comparison between FGSC2489 and FGSC4830. Additionally, 99 gene clusters showed higher copy numbers in FGSC2225 relative to FGSC2489. Despite these differences, the two strains shared 129 conserved syntenic blocks, covering approximately 90% of their respective genomes (37.40 Mb in FGSC2225 and 33.33 Mb in FGSC4830). At the nucleotide level, the strains exhibited 921,817 SNPs—nearly double the count found between FGSC2225 and FGSC2489. Small-scale variations were also more frequent, including 100,390 insertions (1.71 Mb) and 79,044 deletions (492 kb). While the total number of InDels was higher than in the FGSC2225 vs. FGSC2489 comparison, the average InDel length was notably shorter (Table S5).

Beyond macroscopic genomic divergence, significant variation exists at the level of protein-coding genes within the *Neurospora* genus. When comparative ortholog analysis was extended to include representative species within the Sordariaceae family, such as *N. crassa*, *N. hispaniola*, *N. tetrasperma*, and *Sordaria macrospora*, the orthologous gene set of *N. crassa* FGSC2225 demonstrated only partial overlap across these taxa (Figure S2b). These findings highlight the critical role of gene turnover and lineage-specific adaptation in shaping the genomic architecture and functional diversity of the Sordariaceae.

### 3.3. Genetic Relationship and Population Structure

**Figure 2:**
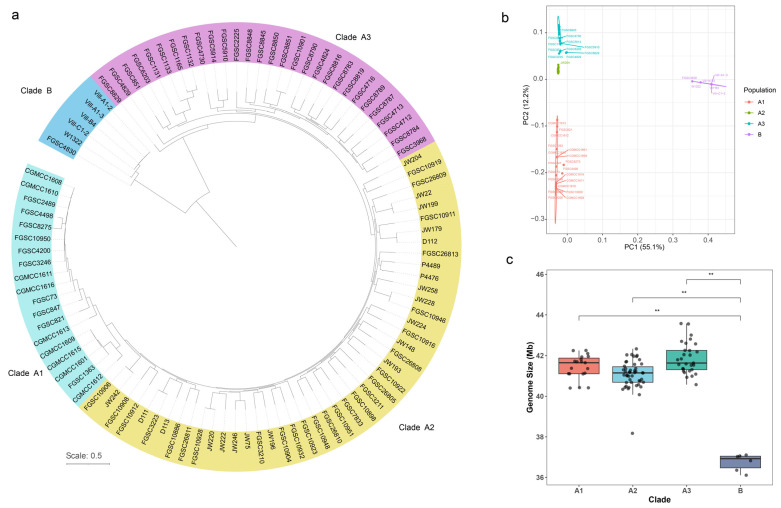
Phylogenomic reconstruction and population structure of *Neurospora crassa*. (**a**) Maximum likelihood phylogenetic tree including 100 *N. crassa* strains. (**b**) Population genetic structure of the global *N. crassa* population. PCA was performed using MAF-filtered and LD-filtered SNPs to minimize bias from rare variants and redundant genomic regions. (**c**) Genome size of *N. crassa* strains. The asterisks (**) indicate a statistically significant difference with *p* < 0.01, as determined by the Wilcoxon rank-sum test.

The phylogenetic analysis of 100 *N. crassa* genomes reveals substantial genetic diversity and clear population stratification (Figure 2a). The population is broadly divided into two primary lineages, designated Clade A and Clade B. Clade B comprises six strains sampled from France and Ivory Coast—including FGSC4380, W1322, Vill-A1-2, Vill-A1-3, Vill-B4, and Vill-C1-2—representing a highly distinct evolutionary lineage (Figure 2a,b). Notably, the genomic sequence divergence on collinear sequences between Clade B and the primary population, Clade A, exceeds 2.4%. To further resolve the population structure, Clade A can be classified into three distinct subclades: Clade A1, comprising 19 strains including FGSC2489; Clade A2, containing 46 strains such as JW222; and Clade A3, which includes 29 strains such as FGSC2225 (Table S6). Additionally, Clade B is characterized by a significantly smaller genome size than Clade A (Figure 2c), consistent with previous reports [33,34].

Based on the FGSC2225 reference genome, 4,877,053 variants were identified across 100 *N. crassa* strains, comprising 3,990,223 SNPs, 511,624 insertions, and 375,206 deletions. A total of 401,442 missense variants were identified, representing 39.39% of the coding potential. To determine whether LoF polymorphisms—such as frameshifts and premature stop codons—contribute to evolution, the SNP and indel datasets were analyzed. In Clade A and Clade B, 4784 and 1214 premature stop codons (stop-gain) and 31,808 and 8773 frameshift variants were identified, respectively. Additionally, 354,029 and 99,990 missense SNVs were recorded (Table S7). By aggregating stop-gain, frameshift, and missense mutations, 31,787 LoF variants were identified across 4859 genes in Clade A, and 8820 LoF variants across 2924 genes in Clade B (Table S8). The dataset of potentially deleterious mutations and LoF provides a valuable resource to study the genetic redundancy within the *N. crassa* genome.

### 3.4. Genome-Wide Identification of TEs in N. crassa

**Figure 3:**
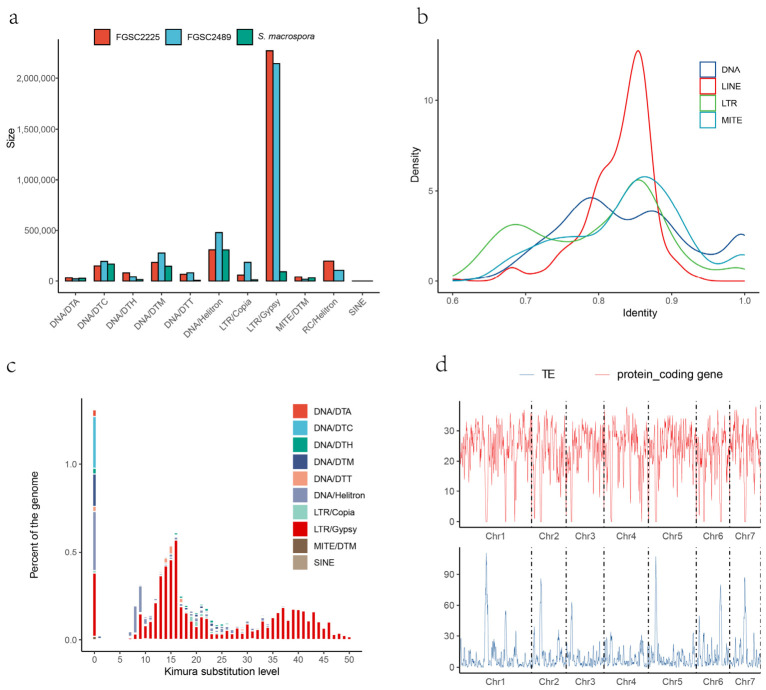
Genome-wide identification of TEs in *Neurospora crassa* FGSC2225. (**a**) The total size of TEs in *N. crassa* FGSC2225 and FGSC2489 is significantly larger than in the RIP-defective close species *S. macrospora*. (**b**) Identity of homologous TEs in FGSC2225. (**c**) Kimura substitution level of TEs in *N. crassa* FGSC2225. (**d**) Genomic distribution of TEs, showing a negative correlation with protein-coding gene density.

A total of 4598 TEs were identified in the FGSC2225 genome (accounting for 11.38% of the total size), a proportion highly consistent with that of the FGSC2489 strain (4471 TEs; 11.37%) (Table 1). Within the Class I retrotransposons, the Long Terminal Repeat (LTR) family was the most abundant in *N. crassa* (7.86%). This group is dominated by the LTR/Ty3 superfamily at 5.87%, while LTR/Ty1 elements represent a significantly smaller fraction at only 0.17%. Following LTRs, LINE elements represented the second largest Class I category at 1.80%, whereas SINE elements were rare, comprising only 0.02%. In addition, the genome of *N. crassa* contains a significantly higher proportion of Class II DNA transposons compared to related species, occupying approximately 2.47% of the FGSC2225 genome (Table 1, Figure 3a). In contrast, the closely related species *Sordaria macrospora* (RIP-absent) possesses significantly fewer TEs (2.63% of the genome)—roughly one-fourth of the proportion observed in *N. crassa*. This discrepancy is primarily driven by the lower abundance of LTR/Ty3 and LINE retrotransposons in *Sordaria macrospora*, whereas its DNA transposons are only slightly lower.

TE content is a major driver of genome size variation within the genus *Neurospora* [35]. In our comparison, we found the genome of FGSC2225 to be 4.04 Mb larger than that of FGSC4830. Increased TE accumulation in FGSC2225 accounted for 3.00 Mb (74.27%) of this expansion, while non-TE regions contributed only 1.04 Mb. A detailed classification of these elements revealed that the FGSC4830 genome contains only 500 kb (1.35%) of LTR/Ty3 retrotransposons, significantly less than the 2.42 Mb (5.87%) identified in FGSC2225. Similarly, DNA transposons occupy only 473 kb (1.28%) of the FGSC4830 genome, compared to 1.02 Mb (2.47%) in FGSC2225.

Evolutionary analysis of diverse TEs revealed mutation patterns unique to *N. crassa*. In the FGSC2225 genome, the sequence similarity of homologous TEs is primarily distributed around 85%, with LINE elements exhibiting a particularly concentrated similarity range (Figure 3b). The Kimura genetic distance for LTR/Gypsy-type transposons shows a characteristic bimodal distribution, with peaks located near 15 and 40 (Figure 3c).

This phenomenon is likely attributable to the RIP mechanism in *N. crassa*, which drives an exceptionally rapid evolutionary rate in TEs, resulting in mutation accumulation rates that are several orders of magnitude in some filamentous fungi higher than those observed in plants or animals. Due to the high variability of TEs across species and environmental conditions, precise estimation of mutation rates remains challenging; consequently, traditional methods of inferring divergence times based on TE sequence divergence in ascomycetes are not applicable in this study.

Further analysis indicated that TEs are widely distributed across all chromosomes in *N. crassa*, displaying a significant negative correlation with the distribution patterns of coding genes (Figure 3d). This spatial genomic architecture likely reflects the long-term evolutionary interplay between transposons and the host genome, while also suggesting a critical role for the RIP mechanism in maintaining genomic stability. When reconstructing the evolutionary history of TEs, species-specific genetic regulatory mechanisms, such as RIP, must be considered as key factors.

### 3.5. Pangenomic Analysis Reveals Complex Structural Variations Across N. crassa Genomes

In this study, by integrating 4 chromosome-level genomes into a graph-based pangenome, we identified a total of 1,157,710 genomic variations, comprising 988,604 SNPs and 174,132 indels (including 86,274 insertions and 87,662 deletions), alongside 690 large-scale presence–absence variations (PAV > 50 bp). This optimized framework can significantly enhance variant calling accuracy by mitigating the inherent limitations of linear reference bias. Unlike traditional mapping, where divergent reads often fail to align, this variation graph provides a topological space that incorporates known allelic diversity as predefined paths. By remapping sequencing data to this graph-encoded transition space, researchers can achieve superior alignment sensitivity in polymorphic regions and more reliable genotyping of SVs and PAVs.

**Figure 4:**
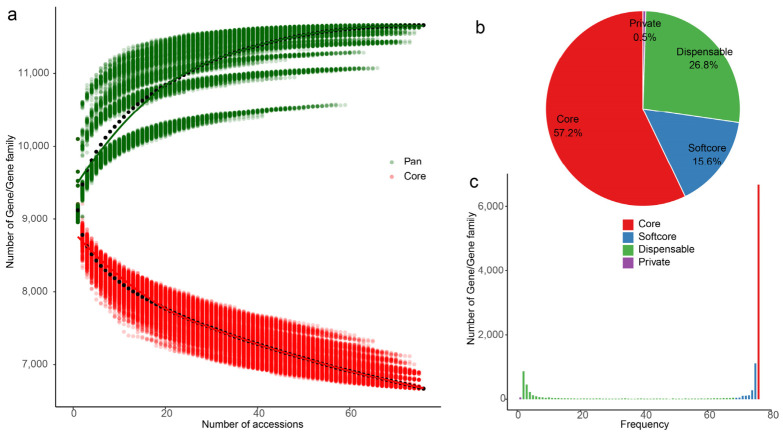
Pan- and core genome analyses of 76 *Neurospora crassa* strains. (**a**) Variation in gene families in the pan-genome and core genome. The pangenome was constructed by 76 *N. crassa* genomes. (**b**) Compositions of the pan-genome and individual genomes. Pie shows the proportion of the gene family marked by each composition. (**c**) Histogram shows the number of gene families in the 76 genomes with different frequencies.

Moreover, we conducted a pangenome analysis of 76 high-quality de novo assembled genomes (draft genome sequence N50 > 100 kb) to explore the molecular resources of *N. crassa*. Orthologs investigation classified all genes from the 76 *N. crassa* genomes into 11,661 families. Of the total gene sets, 6666 (57.2%) families presented in all 76 strains and were defined as core genes, 1814 (15.6%) families presented in 68 to 75 strains (>90% of the collection) were defined as softcore genes, 3126 (26.8%) families presented in 2 to 67 strains were defined as dispensable genes, and 55 (0.5%) families presented in only one strain were defined as private genes (Figure 4 and Figure S3, Table S9). Although the total of dispensable and private gene families accounted for a larger proportion (42.4%) of the total gene sets, they accounted for an average of 27.3% of the genes in individual strains.

### 3.6. N. crassa Pangenome as a Framework for Identifying and Studying Gene Families

The pangenome provides a robust framework for studying the dynamic evolution of gene families. Out of 4765 gene families identified, 1359 contained an average of more than two members per strain (Table S10). Using the Fano factor to measure overdispersion, we identified the most evolutionarily divergent gene families. These included DUF1720 (PF08226.17), DUF7924 (PF25545.2), S1 (PF00575.30), and various Ankyrin repeat families (e.g., PF12796.14, PF00023.37, PF13637.13). While many of these highly divergent families remain unannotated, several functionally characterized domains also ranked highly, such as RNase_H (PF00075.31), HET (PF06985.18), zf-CCHC (PF00098.29), and zf-C2H2_AcuF (PF26082.1), alongside other DNA- and RNA-binding domains.

The HET domain is a fungus-specific sequence approximately 200 amino acids in length, characterized by three conserved blocks of 15 to 30 amino acids [36]. Many HET-domain proteins also contain WD repeat domains composed of a variable number of WD40 units [37]. While 55 HET-encoding genes were previously annotated in the *N. crassa* reference genome, extensive polymorphism has been observed between alleles [20]. Given the vital role of HET domains in filamentous fungi, we utilized our pangenome for a genome-wide identification and evolutionary analysis of this family across the species.

**Figure 5:**
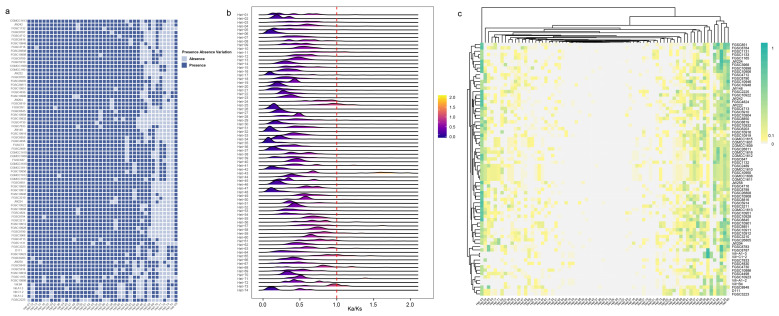
Characterization of HET-domain genes across 76 *Neurospora crassa* strains. (**a**) Presence and absence profiles of the HET-domain gene family within the *N. crassa* pangenome, illustrating gene content diversity across the population. (**b**) Distribution of Ka/Ks values for HET-domain containing genes across *N. crassa* strains. The *x*-axis denotes the range of Ka/Ks values, while the peak height and color density represent the frequency of gene accumulation within specific evolutionary rate intervals for each strain. (**c**) Hierarchical clustering heatmap showing the distribution of genes with Ka/Ks > 1. Strains and genes are clustered based on these positive selection profiles to highlight shared evolutionary patterns across the population.

Across the 76 *N. crassa* strains, we identified 74 HET-domain-containing genes. This family exhibited significant PAV across the population (Figure 5a). For instance, the reference strain FGSC2489 contains two copies of the *Het-01* gene (NCU09954 and NCU11211), whereas FGSC2225 lacks *Het-01* entirely. The distribution of these absences appears strongly linked to phylogeny; for example, *Het-66* was absent in all five strains of Clade B, while *Het-12*, *Het-60*, *Het-61*, *Het-65*, *Het-71*, and *Het-72* were missing specifically in the four Vill strains within Clade B. These findings suggest that HET-domain genes may have diverged functionally between the major clades.

Evolutionary analysis showed that most HET-domain genes have a Ka/Ks ratio below 1, highlighting strong purifying selection to maintain functional protein sequences across the population. In contrast, *Het-24*, *Het-25*, *Het-64*, and *Het-73* exhibited ratios near 1, suggesting potential neutral selection (Figure 5b). Notably, specific genes such as *Het-11*, *Het-25*, and *Het-59* contained multiple copies with Ka/Ks > 1 in certain strains, indicating potential localized adaptive evolution (Figure 5c).

Among these candidates, *Het-26* (NCU03493) and *Het-35* (NCU03125) are part of the nonallelic het-C/het-E incompatibility system, which is the best-characterized system in *N. crassa* [38] and *Podospora anserina* [37]. Previous studies noted extreme diversity at these loci; for example, het-6 alleles in *N. crassa* are only 68% identical [36], and het-C alleles contain highly divergent regions that define specificity [38]. While these loci have been reported to be under balancing selection [39], our pangenome-wide analysis found their Ka/Ks ratios to be below 0.2 (Figure S5), suggesting they are currently undergoing purifying selection across the broader population.

### 3.7. N. crassa Pangenome as a Framework for Identifying and Studying Stress-Related Gene Families

The dynamic expansion of transcription factor (TF) families reflects the evolving regulatory complexity of fungal genomes. In fungal species, TFs such as bZIP protein orthologs play critical roles in vegetative growth, sexual and asexual development, stress response, secondary metabolite production, and virulence both in human pathogens [40]. Within Ascomycetes lineages, specific methylation architectures may drive the notable expansion and evolutionary maintenance of these broadly distributed regulatory landscapes, including the bZIP, GATA, HLH, Homeobox, and heat shock transcription factors (HSFs) families [41].

To facilitate the genome-wide identification and evolutionary analysis of such functional genes in *N. crassa*, the pangenome was utilized as a robust framework to shed light on the molecular evolution of various gene families. Here, we conducted a systematic identification and phylogenetic analysis of three key gene families: *HSF*, *bZIP*, and *Cytochrome P450* (*CYP450*).

Adaptation to thermal stress, a critical area of fungal research, is primarily mediated by HSF proteins in eukaryotes. These factors regulate the expression of heat shock proteins (HSPs) that function as molecular chaperones. For instance, *Neurospora* hsp30-based promoters represent a new set of modular elements serving as transcriptional rheostats to adjust gene expression or implement regulated circuitries for synthetic biology and biotechnological strategies [42]. Given this importance, we conducted a targeted analysis of the HSF transcription factor family. Domain searches identified two HSF proteins (NCU08512 and NCU08480) containing complete HSF DNA-binding domains, while RRG-2 (NCU02413) encodes a protein with a truncated HSF DNA-binding domain in FGSC2489. Phylogenetic analysis across the pangenome demonstrated that all 76 strains harbor exactly three copies of these HSF proteins, indicating they are essential core components of the *N. crassa* genome (Figure S4, Table S11).

In contrast to the highly conserved *HSF*s, the *bZIP* and *CYP450* families exhibit greater population-level diversity. While 10 *bZIP* genes were identified in the majority of *N. crassa* strains, we detected PAVs in five *bZIP* genes across seven specific strains (Figures S5 and S6). Similarly, *CYP450* genes were classified into 39 distinct groups through phylogenetic and paralog analysis. Notably, all strains within Clade B lack the *CYP450-35* and *CYP450-36* paralogs (Figures S7 and S8). This clade-specific loss suggests potential functional divergence or specialized metabolic requirements within this lineage, which prefer further functional research.

**Figure 6:**
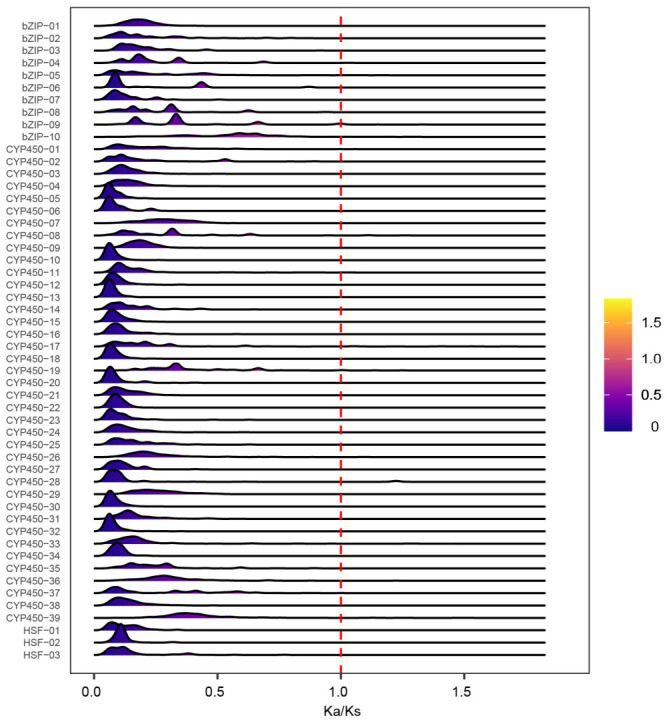
Ka/Ks distribution. Distribution of Ka/Ks values of *HSF*, *bZIP*, and *CYP450* genes in 76 *Neurospora crassa* strains. The *x*-axis represents the range of the Ka/Ks values, while the height and color of the peaks in the figure indicate the number of gene accumulations in different *N. crassa* strains.

Interestingly, the overall extent of PAV within these three stress-related gene families in *N. crassa* appears lower than that typically observed in plant and animal pangenome studies. This relative stability may be attributed to the RIP mutation mechanism, which effectively maintains genes in a single-copy state by targeting and mutating duplicated sequences. Furthermore, Ka/Ks values for the genes peak below 0.5, the Ka/Ks distributions except for *bZIP-10*. This indicates that they are primarily under purifying selection (Figure 6), highlighting a strong purifying pressure to maintain functional protein sequences across the population.

## 4. Discussion

Accurate and high-quality genome assembly and annotation are fundamental to advancing genomic research. As a high-quality alternative reference, the FGSC2225 genome provides a robust platform for characterizing variation across *Neurospora* lineages. In this study, we utilized these comprehensive annotations of coding genes and TEs to investigate the molecular and evolutionary dynamics of *N. crassa*.

Building on the high-quality assembly of FGSC2225, we constructed two complementary pangenome frameworks for *N. crassa*, a graph-based pangenome and a population-scale pangenome. The pangenome concept represents a major breakthrough in plant genomics, providing a comprehensive foundation for harnessing genetic diversity and fast-tracking crop improvement [43]. To date, the pan-genomes of four model fungal species have been reported, including *Saccharomyces cerevisiae*, *Agaricus bisporus*, *Candida albicans*, *Cryptococcus neoformans*, *Aspergillus fumigatus*, *Pyrenophora tritici-repentis*, and *Zymoseptoria tritici* [6,44,45,46]. Furthermore, dedicated pangenome databases have been constructed to facilitate the comparative analysis of these complex datasets [47,48].

The construction of the *N. crassa* pangenome provides a high-resolution map of genomic diversity, enabling the strategic selection of gene candidates for functional validation. This pangenomic approach is broadly applicable across fungal species, providing a blueprint for understanding evolutionary success. By categorizing the gene repertoire into core and accessory components, we can distinguish between essential regulatory elements and those driving lineage-specific adaptation. Specifically, this framework identifies strains with unique genetic backgrounds, such as Clade B lineages that naturally lack the *CYP450-35* and *CYP450-36* genes. These strains serve as ideal candidates for experimental crosses and transgenic validation, facilitating a deeper exploration of gene function.

Filamentous fungi spontaneously undergo vegetative cell fusion events (anastomoses) within and between individuals, leading to cytoplasmic mixing and the formation of vegetative heterokaryons containing different nuclear types. The viability of these heterokaryons is genetically controlled by specific loci termed *het* loci. Heterokaryotic cells formed between individuals with incompatible het genotypes undergo a characteristic cell death reaction or suffer severe growth inhibition. Genes defining vegetative incompatibility are highly polymorphic. Most, if not all, incompatibility systems include a protein partner bearing the fungus-specific HET domain. This domain acts as the mediator of cell death; a modular conception of these systems suggests that recognition is ensured by the variable regions of incompatibility proteins, while the HET domain triggers the actual cell death response. Previous reports mainly focused on the study of het genes highly related to the vegetative cell fusion events, which were only a small part of the HET domain containing genes. In this study, by utilizing the *N. crassa* pangenome, we expanded our scope to identify all HET-domain-containing genes across the *N. crassa* population. We analyzed their evolution, contributing to a broader understanding of how cell death mediators evolve in filamentous fungi.

Ultimately, these findings demonstrate that the pangenome is not merely a catalog of variation but a predictive tool that streamlines the transition from evolutionary bioinformatics to experimental functional genomics. Since many filamentous fungi are significant plant pathogens, and current genomic resources often lack the resolution to detect their rapid and adaptive evolution, this work addresses a critical gap in fungal genomics.

The methodologies and pangenomic framework established here provide a robust template for studying diverse filamentous fungi, particularly those where pangenome-scale analyses are essential for identifying effectors, host-adaptation genes, and structural variations linked to stress and virulence. By moving beyond a single reference genome, this approach allows for a more comprehensive understanding of the genetic factors that contribute to fungal fitness and pathogenicity in ever-changing environments.

## 5. Conclusions

This study establishes a comprehensive pangenomic framework for *Neurospora crassa* by integrating a high-quality assembly of the distinct isolate FGSC2225 with global strain datasets. The results confirm a deep evolutionary divergence that splits the population into two primary lineages, Clade A and Clade B, with genome size variations driven by transposable element accumulation and repeat-induced point mutations. Additionally, the graph pangenome uncovers significant structural plasticity, presence–absence variations, and distinct adaptation patterns in heterokaryon incompatibility and stress-response gene families. This framework enhances our understanding of genetic redundancy and adaptive evolution in filamentous fungi, serving as a valuable resource for future functional genomic research.

## Figures and Tables

**Table 1:** Annotation of repetitive elements in the FGSC2225 genome.

Class	*Neurospora crassa* FGSC2225			*Neurospora crassa* FGSC2489			*Sordaria macrospora*		
	No. of Elements	Length (bp)	Percentage	No. of Elements	Length (bp)	Percentage	No. of Elements	Length (bp)	Percentage
Retroelements	1476	3,243,723	7.86%	1714	2,914,960	7.09%	955	197,594	0.51%
SINEs	112	8749	0.02%	124	9409	0.02%	103	8100	0.02%
LINEs	264	743,805	1.80%	247	465,548	1.13%	270	67,754	0.18%
LTR elements	1100	2,491,169	6.04%	1343	2,440,003	5.94%	582	121,740	0.32%
Ty1/Copia	110	69,598	0.17%	138	222,937	0.54%	114	15,597	0.04%
Ty3/Gypsy	973	2,419,000	5.87%	1205	2,217,066	5.39%	449	104,538	0.27%
DNA transposons	2013	1,017,340	2.47%	2345	1,266,342	3.08%	1942	698,705	1.82%
hobo-Activator	123	94,843	0.23%	169	57,491	0.14%	74	14,585	0.04%
Tc1-IS630-Pogo	126	72,862	0.18%	191	45,389	0.11%	0	0	0
Rolling-circles	87	178,576	0.43%	66	109,066	0.92%	0	0	0
Unclassified	1022	430,692	1.04%	799	378,857	11.09%	621	113,024	0.29%
Total repeats	4598	4,691,755	11.38%	4471	4,671,782	11.37%	3518	1,009,323	2.63%

## Data Availability

All data generated or analyzed during this study are included either in this article or in the Supplementary Information files. The pangenome data of *Neurospora crassa* are available at Zenodo (https://zenodo.org/records/20836033, accessed on 14 May 2026).

## Supplementary Materials

The following supporting information can be downloaded at: https://www.mdpi.com/article/10.3390/jof12070507/s1, Table S1: Main features of *Neurospora crassa* FGSC2225 genome assembly and gene annotation; Table S2: Overview of ncRNAs in *Neurospora crassa* FGSC2225 genome; Table S3: Compared with strain FGSC2489, the unique annotated gene observed in the genome of *Neurospora crassa* FGSC2225; Table S4: Comparison of gene copy number between strains FGSC2225 and FGSC2489; Table S5: Mapping depth and genome coverage of sequenced samples; Table S6: Source and information of 100 *Neurospora crassa* strains; Table S7: Genetic differentiation and diversity within 100 *Neurospora. crassa* strains; Table S8: LoF genes in *Neurospora crassa* population; Table S9: Pan Gene families in *Neurospora crassa* pangenome; Table S10: PAV of gene families in *Neurospora crassa*; Table S11: Pan-gene family of *HSF*, *bZIP* and *CYP450* in *Neurospora crassa*; Figure S1: Genome sequence and gene annotation of *Neurospora crassa* FGSC2225; Figure S2: Intronic divergence in coding genes in relation to protein tertiary structure; Figure S3: Presence and absence information of pan gene families in the 76 *Neurospora crassa* genomes; Figure S4: Phylogenetic tree of *HSF* gene family in *Neurospora crassa*; Figure S5: Presence and absence information of *bZIP* transcription factor gene family in *Neurospora crassa* pan genomes; Figure S6: Phylogenetic tree of *bZIP* gene family in *Neurospora crassa*; Figure S7: Presence and absence information of *CYP450* gene family in *Neurospora crassa* pan genomes; Figure S8: Phylogenetic tree of *CYP450* gene family in *Neurospora crassa*.

## Abbreviations

The following abbreviations are used in this manuscript:

RIP	Repeat-Induced Point mutation
TE	Transposable element
TF	Transcription Factor
bZIP	Basic Leucine Zipper
HSF	Heat Shock Transcription Factors
CYP450	Cytochrome P450
