# Supplementary material for: The Neurospora crassa Pangenome: A Robust Framework for Population-Scale Analysis and Structural Variant Discovery
Source: J Fungi (Basel). 2026 Jul 9;12(7):507. doi: 10.3390/jof12070507 (PMC13412767; doi:10.3390/jof12070507)

Supplementary Material

Figure S1. Genome sequence and gene annotation of *Neurospora crassa* FGSC2225. (a) 7,012 genes annotated in the Swiss-Prot, UniProt, InterProScan, eggnog-mapper and NR databases. (b) Upset diagram showing the overlap of orthologous and orphan genes in *N. crassa* FGSC2225, FGSC2489, FGSC4830; *N. hispaniola*; *N. tetrasperma* FGSC2509 and *Sordaria macrospora*.

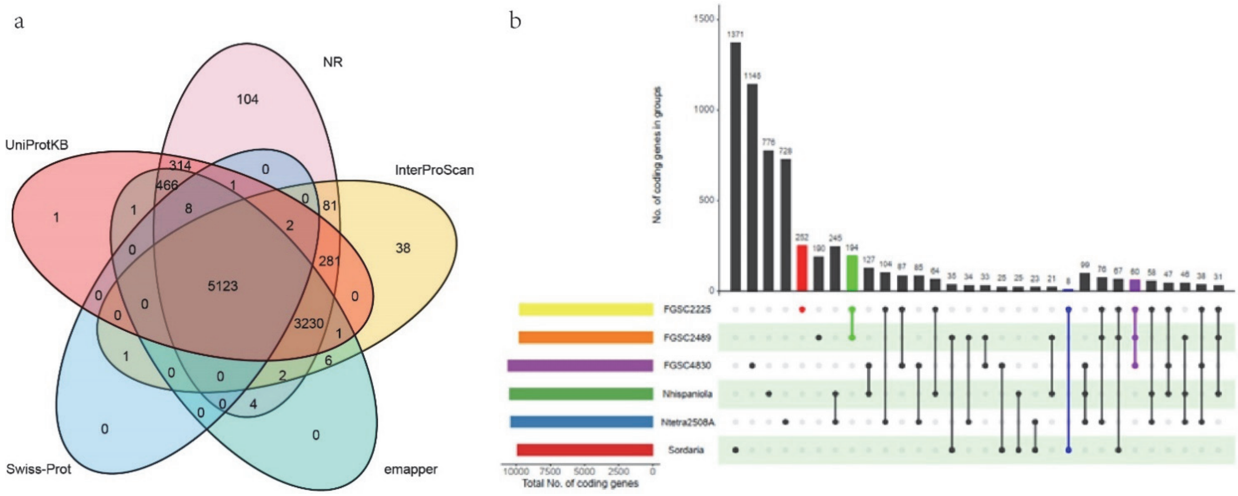

Figure S2. Intronic divergence in coding genes in relation to protein tertiary structure. (a) Comparative analysis of intron counts per gene between homologous loci of *N. crassa* strains FGSC2225 and FGSC2489. (b) Sankey diagram illustrating the conservation, gain, and loss of introns within orthologous gene pairs between FGSC2225 and FGSC2489.

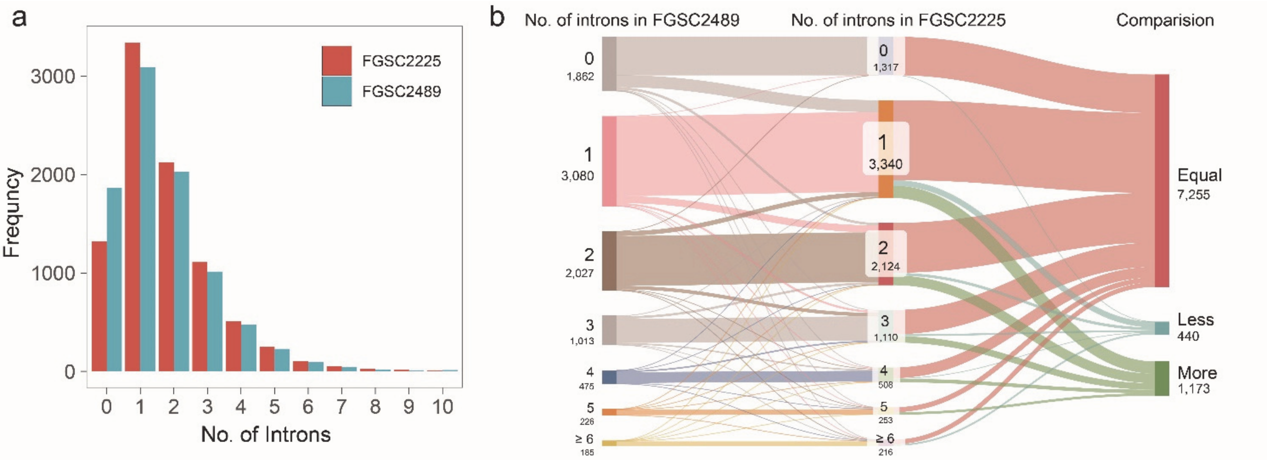

Figure S3. Presence and absence information of pan gene families in the 76 *Neurospora crassa* genomes.

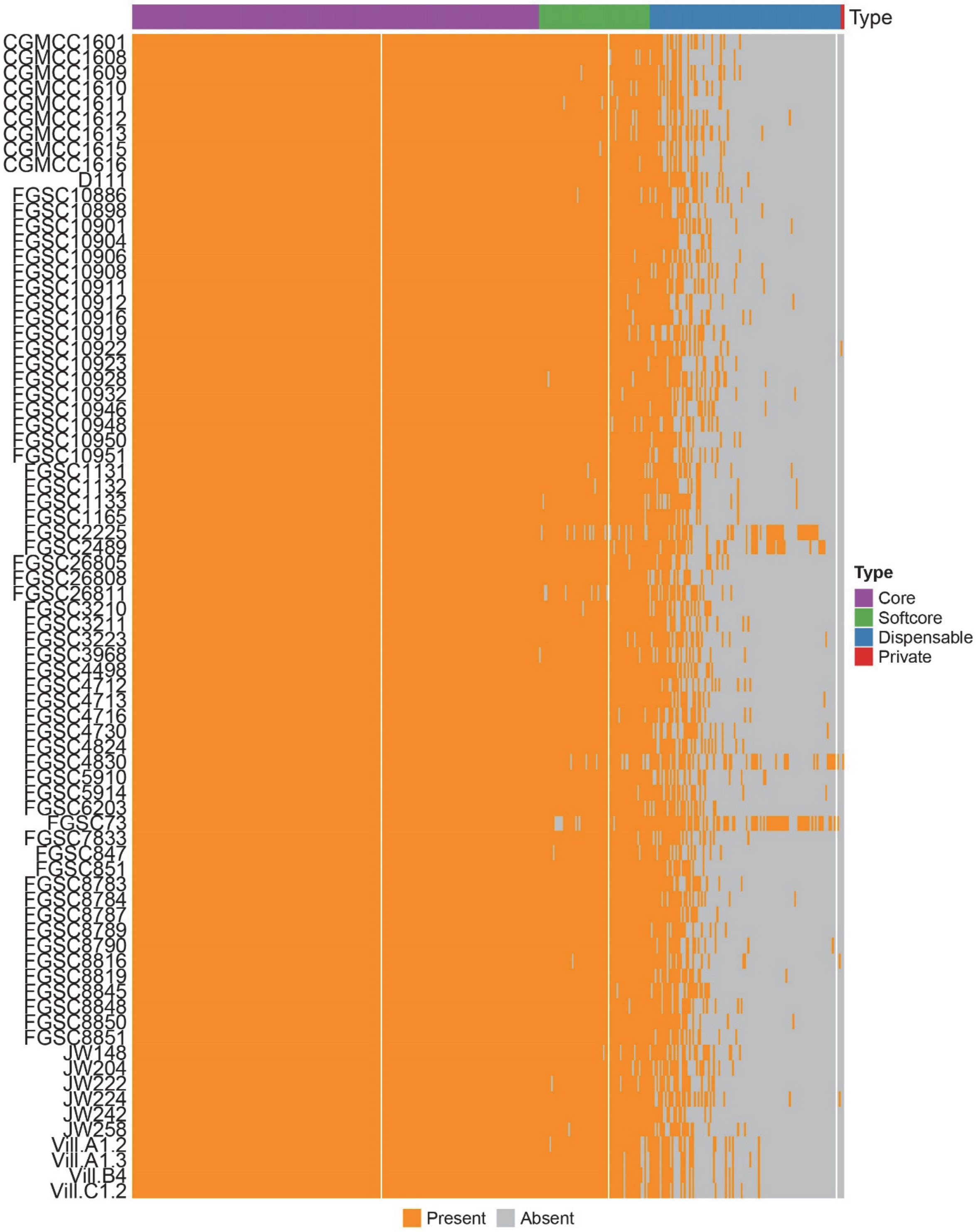

Figure S4. Phylogenetic tree of *HSF* gene family in *Neurospora crassa*.

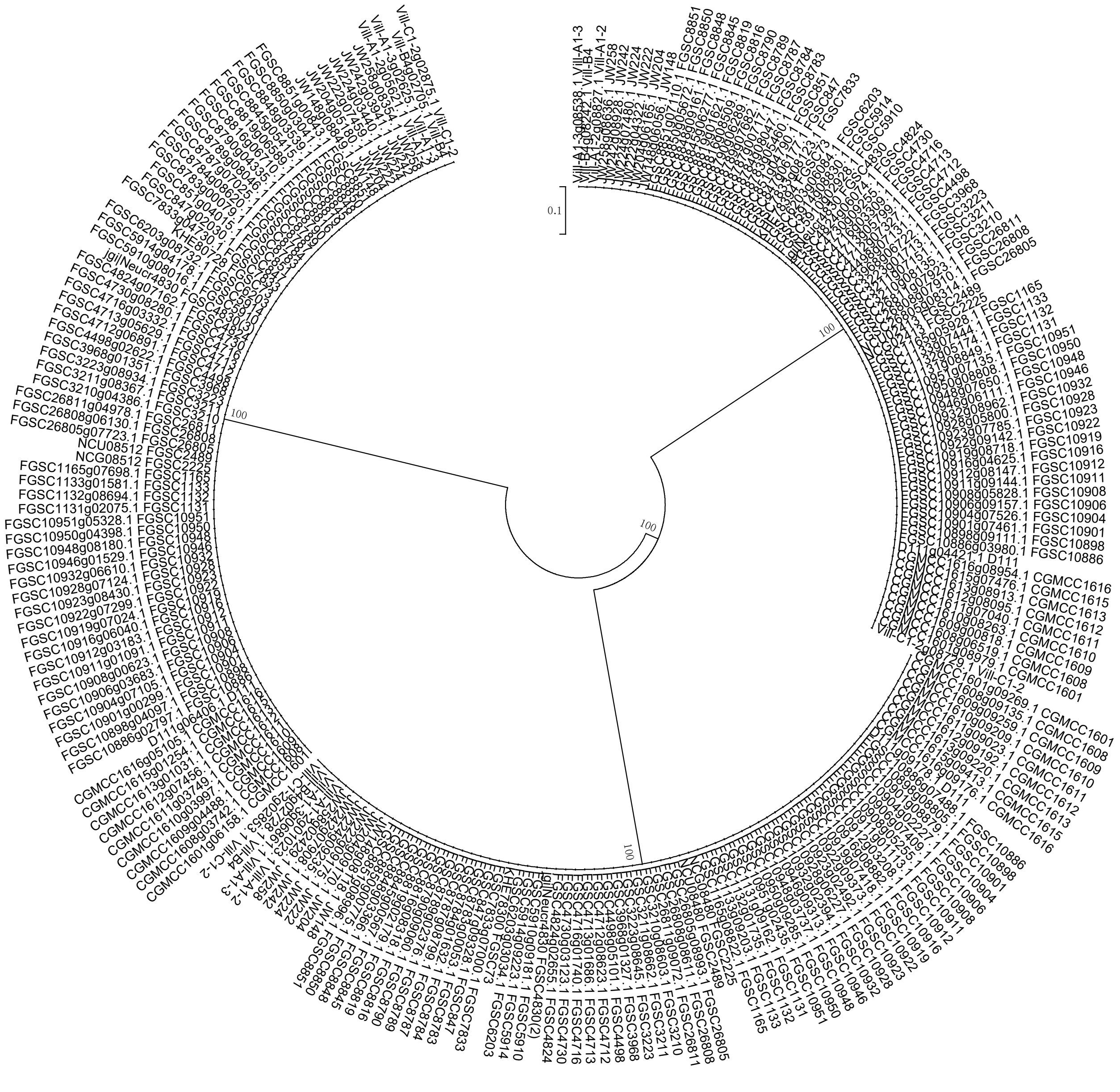

Figure S5. Presence and absence information of *bZIP* transcription factor gene family in *Neurospora crassa* pan genomes.

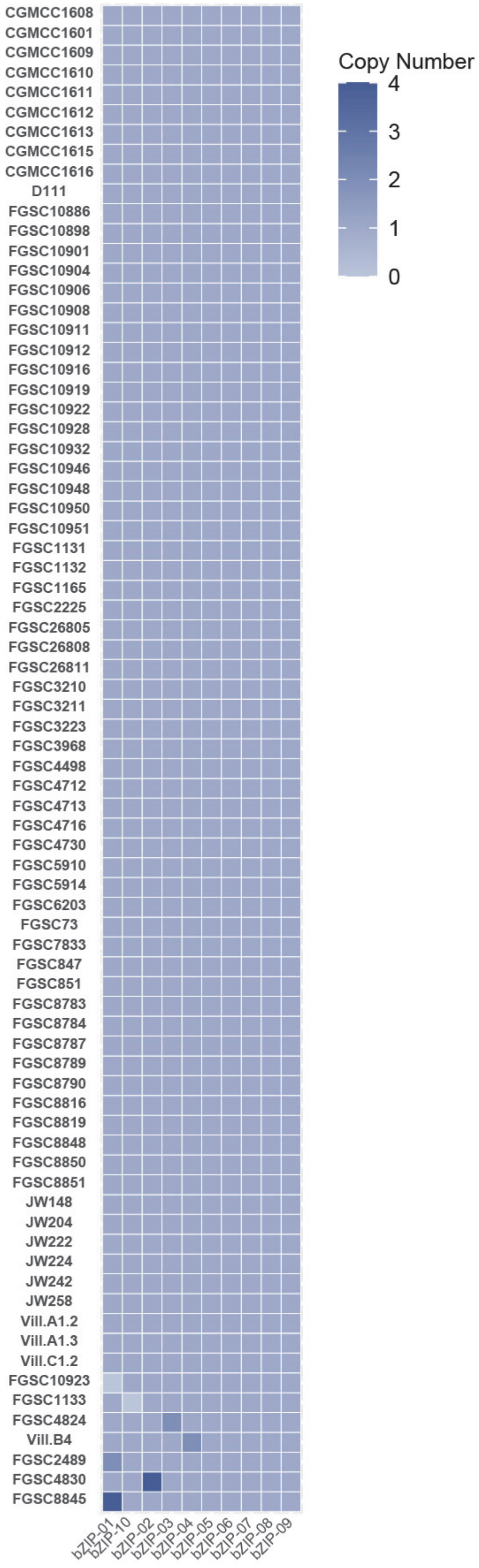

Figure S6. Phylogenetic tree of *bZIP* gene family in *Neurospora crassa*.

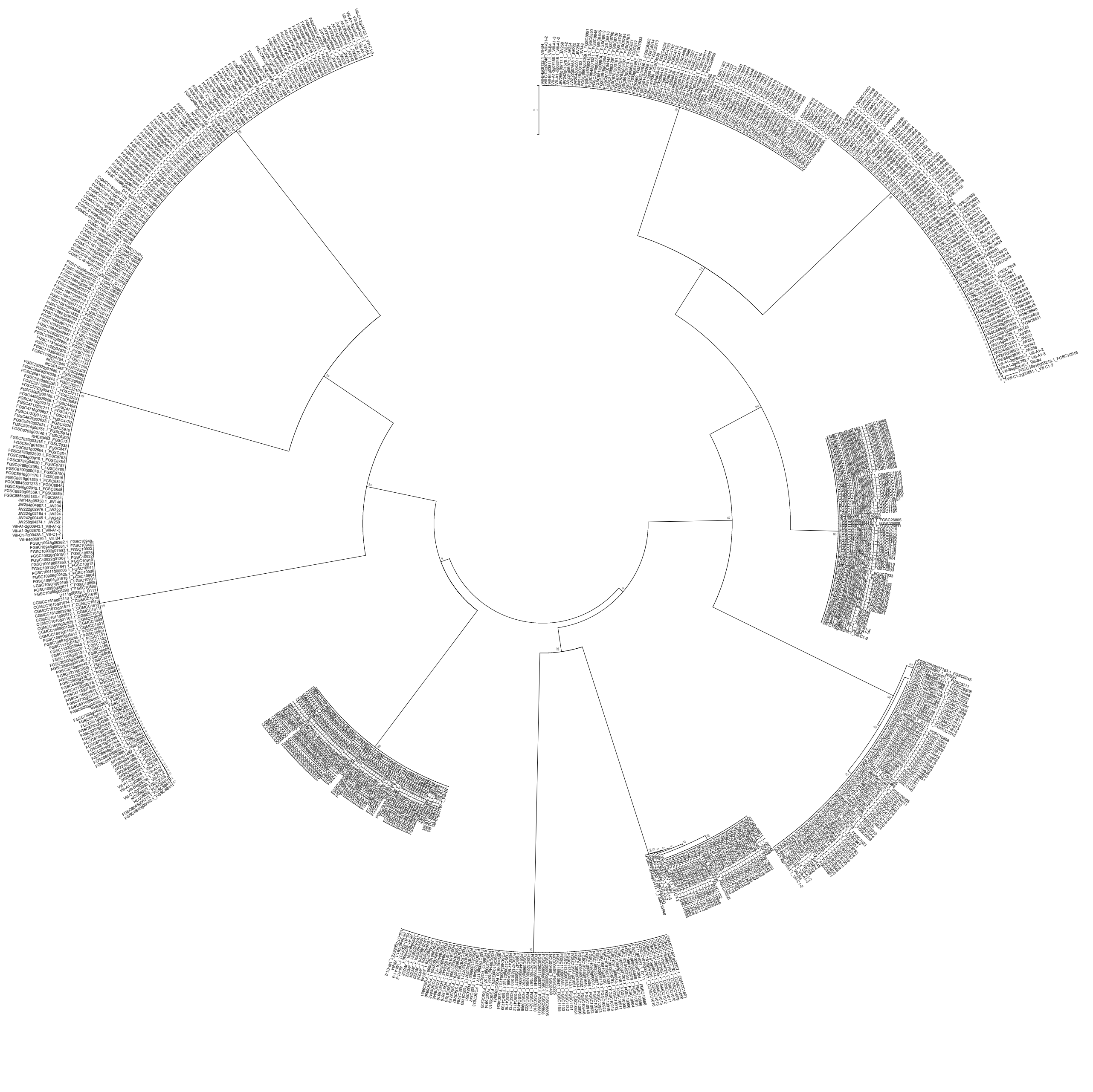

Figure S7. Presence and absence information of *CYP450* gene family in *Neurospora crassa* pan genomes.

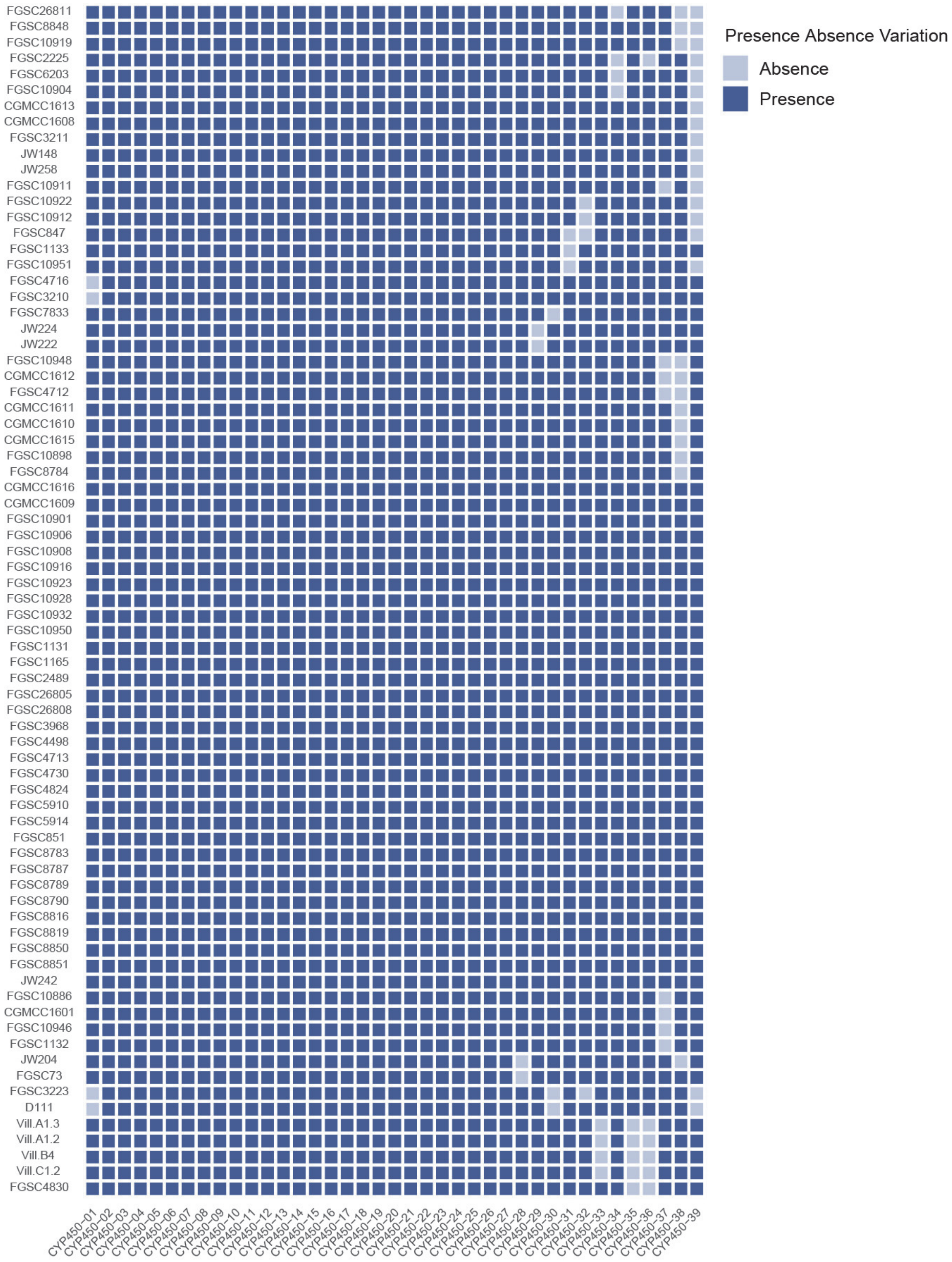

Figure S8. Phylogenetic tree of *CYP450* gene family in *Neurospora crassa*.

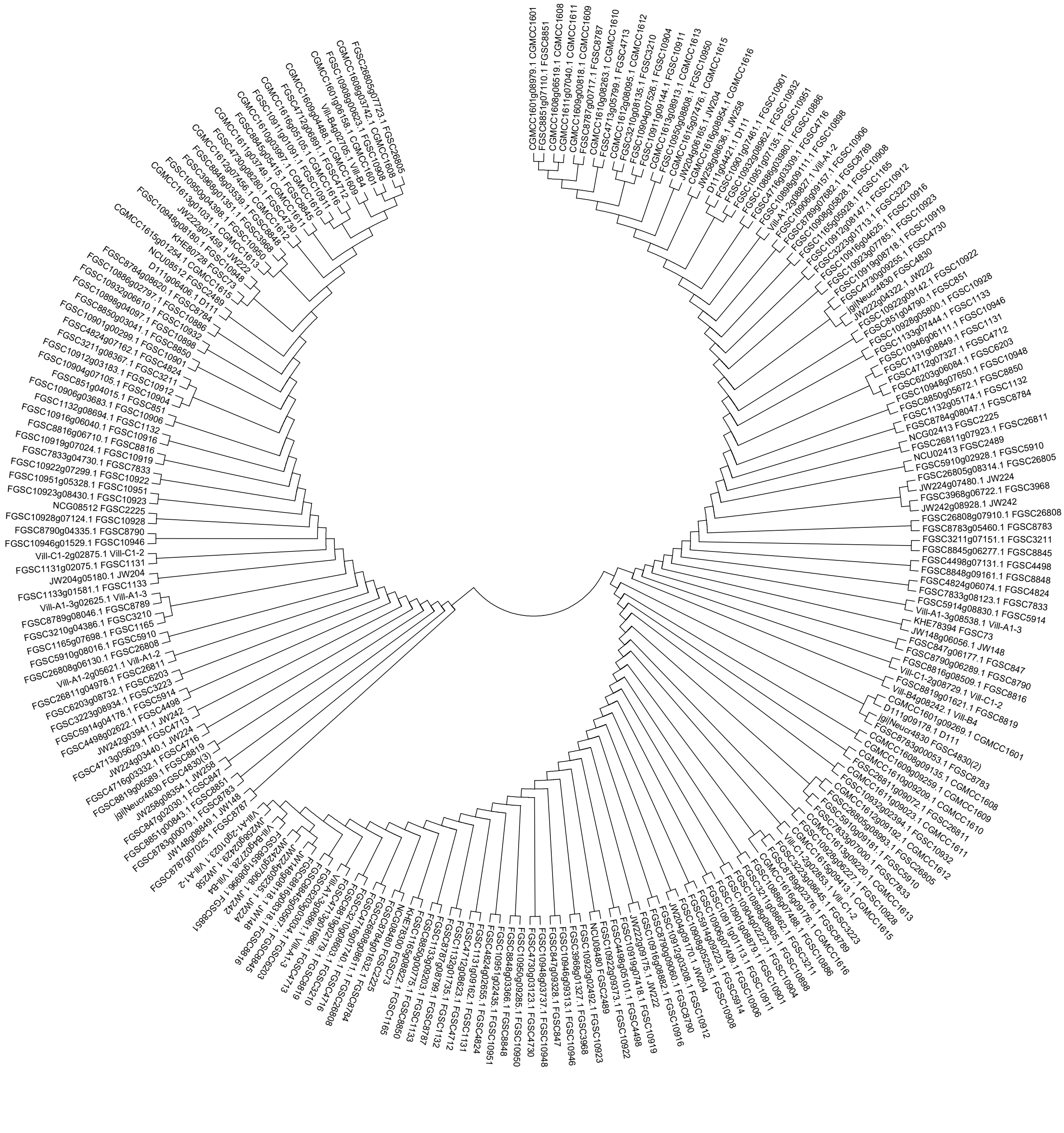

Supplement: Supplementary file 1 [file jof-12-00507-s001.zip › FGSC2225-SupFigures-20260709.pdf]
